# WORKING BACK TO NORMAL FUNCTION FOLLOWING HOSPITALIZATION: A GROUNDED THEORY STUDY

**DOI:** 10.1093/geroni/igz038.1671

**Published:** 2019-11-08

**Authors:** Daniel Liebzeit, Lisa Bratzke, Barbara King

**Affiliations:** 1 University of Wisconsin-Madison School of Nursing, Madison, Wisconsin, United States; 2 University of Wisconsin-Madison, Madison, Wisconsin, United States

## Abstract

Transitions older adults experience post hospital discharge have primarily focused on the process of moving care from one setting to another (e.g. hospital to home). Older adults often experience a significant transition in terms of losing functional status after a hospital stay. Little is known about how older adults regain their functional ability, the type of work they engage in to recover, and conditions that influence their ability to work after a hospital stay. The objective of this Grounded Theory study was to understand strategies older adults use post discharge as they work to regain their functional status and what conditions facilitate or limit their ability to work toward returning to normal. A qualitative study was conducted. Adults aged 65 and older discharged from a large Midwestern teaching hospital (N = 14) were interviewed using in-depth one-on-one interviews. Data were analyzed using open, axial, and selective coding. Participants described key strategies they employed to regain their normal function following hospitalization and illness: doing exercises, expanding physical space, resuming prior activities and daily cares, and tracking improvement with benchmarks. Several conditions such as, presence of informal (family, friends) and formal (healthcare providers) support, perceived threats (relocation), and having poor physical or physiologic function, acted as barriers and facilitators to participants ability to work back to normal function. This study provides empirical data on work older adults engage in to transition back to normal function during the post discharge period. It presents opportunities for better supporting their work of regaining function.

